# Association of *estrogen receptor beta* variants and serum levels of estradiol with risk of colorectal cancer: a case control study

**DOI:** 10.1186/1471-2407-12-276

**Published:** 2012-07-03

**Authors:** Huanlei Wu, Li Xu, Jigui Chen, Junbo Hu, Shiying Yu, Guangyuan Hu, Liu Huang, Xiaoping Chen, Xianglin Yuan, Guojun Li

**Affiliations:** 1 Department of Oncology, Tongji Hospital, Huazhong University of Science and Technology, 1095 Jiefang Ave, Wuhan, 430030, Hubei, China; 2 Department of Surgery, Tongji Hospital, Huazhong University of Science and Technology, 1095 Jiefang Ave, Wuhan, 430030, Hubei, China; 3 Department of Surgery, Wuhan 8th Hospital, Wuhan, People’s Republic of China; 4 Department of Head and Neck Surgery, The University of Texas MD Anderson Cancer Center, Houston, TX, USA; 5 Department of Epidemiology, The University of Texas MD Anderson Cancer Center, Houston, TX, USA

**Keywords:** Colorectal cancer, Endogenous estrogen, Estrogen receptors, Single nucleotide polymorphisms

## Abstract

**Background:**

Endogenous estrogens may play a vital role in colorectal tumorigenesis. Estrogen receptor beta is the predominant subtype which mediates the biological effect of estrogens, while loss of expression of estrogen receptor beta has been indicated as a common step in the development of colorectal cancer (CRC). Epidemiological studies have revealed several functional polymorphisms of *estrogen receptor beta* (*ESR2*) for cancer risk, but relevant study in CRC is limited, particularly in men. This study aimed to investigate the association of circulating estradiol and variations of *ESR2* with CRC risk in men.

**Methods:**

We initiated a case–control study consisting of 390 patients with CRC and 445 healthy controls in men only. We genotyped *ESR2* single nucleotide polymorphisms (SNPs) rs1256049 and rs4986938 and measured serum estradiol concentration using chemilluminescence immunoassay. Multivariable logistic regression model was performed to evaluate the associations between these variables and CRC risk.

**Results:**

*ESR2* rs1256049 CT/TT genotypes were associated with reduced risk of CRC (odds ratio [OR], 0.7, 95% confidence interval [CI], 0.5–1.0), while rs4986938 CT/TT genotypes were associated with increased risk of CRC (OR, 1.5, 95% CI, 1.0–2.1). In addition, the CRC risk increased with the number of risk genotypes of these two SNPs in a dose–response manner (*P*_*trend*_, 0.003). Specifically, subjects carrying risk genotypes of both SNPs had the highest risk of CRC (OR, 2.0, 95% CI, 1.3–3.3.). Moreover, serum estradiol concentration alone was associated with risk of CRC in men (OR, 1.2, 95% CI, 1.0–1.3). However, individuals presenting both rs4986938 CT/TT genotypes and high level of serum estradiol had a high risk of CRC (OR, 2.3, 95% CI, 1.4–3.9), compared with those presenting CC genotype and low level of serum estradiol. The similar joint results were not observed for SNP rs1256049.

**Conclusions:**

These results suggest that endogenous estrogen and genetic variations in *ESR2* may individually, or more likely jointly, affect CRC risk in male Han Chinese population, while larger studies are needed to validate our findings.

## Background

Colorectal cancer (CRC) is a major health problem in China and worldwide. With more than one million new cases diagnosed and more than 500,000 deaths each year, it has become the third most common cancer and the fourth most common cause of cancer death globally [1,2]. In addition to smoking, alcohol drinking, dietary factors and obesity, estrogens appear to play a role in CRC carcinogenesis [3-5]. Inherited predisposition may also influence CRC development [1].

While typically referred to as female hormone, estrogens regulate a board range of important functions in both females and males. Estrogen induction of cell proliferation has been considered as a critical step in carcinogenesis of gynecologic tissues including breast, endometrium and ovary, and also has been suggested to involve in cancer of colon and prostate [6]. An emerging body of evidence supports that endogenous and exogenous estrogens may play different roles in colorectal tumorigenesis [7]. For instance, the Women’s Health Initiative (WHI) study observed reduced CRC rates by 37% in postmenopausal women receiving estrogen-plus-progestin formulations compared to those receiving placebos, and several studies have reported similar observations [8-12]. In contrast, two prospective studies reported that high concentrations of circulating estrogen conferred increased risk for CRC [13,14]. It is noted, however, that all the studies published used women only as the study subjects. The correlation between endogenous estrogen and the risk of CRC in men has not yet been evaluated.

The biological effects of estrogens are mediated by estrogen receptors (ER) *α* and *β*, which belong to the nuclear hormone receptor superfamily and act as ligand-activated transcription factors [15]. ERβ is the predominant subtype of estrogen receptor in colorectal epithelial cells; its loss of expression has been indicated as a common step in the development of CRC [6,16-18]. ERβ is encoded by *estrogen receptor β* (*ESR2*) gene, which is located on chromosome 14q23.2 and covers a genomic region of 111.5 kb [19]. Several *ESR2* genetic variants have been investigated, including rs1256049 in exon 5, which causes a synonymous change of unknown functional significance, and rs4986938, which is located in the 3′-untranslated region of the *ESR2* gene [20,21]. Both rs1256049 and rs4986938 have been associated with a number of diseases, including breast cancer, endometrial cancer, prostate cancer, reduced bone mineral density, and cardiovascular disease [22-28]. Only few studies attempted so far to evaluate the ESR2 polymorphism in cancer of colon and rectum and reported conflicting results [25,29].

Given the potential roles of estrogens in CRC and the importance of ERβ in estrogen function, we hypothesized that endogenous estrogen level and *ESR2* genetic variations may individually, or more likely jointly, be associated with the risk of CRC in men. We therefore conducted this male only case–control study to examine the role of functional *ESR2* SNPs rs1256049 and rs4986938 and circulating concentrations of estradiol in CRC risk.

## Methods

### Study population

The present study included a total of 835 unrelated male subjects, of which 390 were patients with CRC and 445 were controls. Among patients with CRC, 168 were recruited between 2008 and 2011 from Tongji Hospital, which is affiliated with Tongji Medical College, and 222 were recruited between 2009 and 2011 from Wuhan Eighth Hospital. These two hospitals were chosen because they are the regional reference center for CRC treatment. Biopsy evaluation was performed in each case and the final diagnosis was confirmed by histopathology. The controls are healthy volunteers recruited from Tongji Hospital Physical Center during the same period who received a comprehensive health examination. The exclusion criteria for both cases and controls included 1) age less than 15 years at the time of diagnosis (cases) or recruitment (control); 2) previous history of malignancies; 3) history of inflammatory bowel disease including familial adenomatous polyposis, ulcerative colitis, and Crohn’s disease; 4) history of chronic hepatic, renal and endocrine disease; 5) family history of CRC; 6) currently taking medication which was known to influence sex hormone level, and 7) diagnosed with hypertension and diabetes mellitus, which could affect sex hormone level. In addition, those who had gastrointestinal pain, detection of blood in stool, or currently diagnosed with communicable diseases such as tuberculosis and acquired immune deficiency syndrome were excluded from the control group. The cases and controls were from the same geographic region and limited to Han Chinese ethnicity.

Eligible participants were interviewed in person to collect demographic and exposure information including smoking and alcohol drinking status (response rate 98%). Participants who have smoked more than 100 cigarettes in their lifetimes were classified as ever-smokers; the others were classified as never-smokers. Participants who have drunk alcohol beverage at least once a week for more than one year were classified as ever-drinkers; the others were classified as never-drinkers.

The study conformed to guidelines set forth by the Declaration of Helsinki and was approved by the ethics committee of the Tongji Medical College. Written informed consent was obtained from each participant before recruitment.

### Genotyping

Peripheral blood samples were collected from all participants. Genomic DNA was extracted from peripheral blood using a FUJI whole blood DNA kit (Fujifilm Corporation, Japan). All participants were genotyped for rs1256049 and rs4986938 polymorphisms using a Taqman SNP genotyping assay implemented by the ABI Prism 7900HT Sequence Detection System (Applied Biosystems). Primer and probe sequences were optimized using the SNP assay design service provided by Applied Biosystems. For each SNP, the call rate was more than 98%. To verify the accuracy of Taqman genotyping results, 5% of the samples were randomly selected and genotyped using the direct-sequencing method with a concordance of no less than 99%.

### Analysis of serum estrogen concentration

Endogenous estrogens are a group of steroidal compounds including 17b-estradiol, estrone, and estriol. Because the bioactivity of 17b-estradiol is the greatest of the three *in vivo*[30], we chose to evaluate estradiol concentration in serum. Estradiol concentrations were measured using blood samples collected at the initial visit. The blood samples were centrifuged at 1300 *g* for 10 minutes, and the separated sera were stored at −70 °C within 2 hours of blood collection.

Because some samples did not have enough serum for measurement, estradiol concentrations were available from 359 male patients and 378 male controls. The detection rate was 99%. Participants having no estradiol data were not significantly different from participants having estradiol data in regard to age, gender, smoking, or drinking status (data not shown). Using 300 μl of available serum, estradiol concentrations (in pictograms per milliliter) were quantified using sensitive *in vitro* estradiol diagnostic kits (the normal male adult reference value is 20–75 pg/ml) (chemiluminescence immunoassay, Beckman Coulter Inc., USA). Laboratory personnel were blinded to case status. The samples were analyzed in random order, and approximately 5% of the samples were repeated with a 100% concordance.

### Statistical analysis

The distributions of demographic and exposure characteristics among the cases and controls were compared with Pearson chi-square test. For each *ESR2* SNP, deviation from Hardy-Weinberg equilibrium was assessed by chi-square test in controls. Multivariable logistic regression model, with adjustment for potential confounding factors, was used to calculate odds ratios (ORs) and 95% confidence intervals (CIs) for genotype-specific cancer risk. In addition, we conducted subgroup analyses stratified by age, smoking status and alcohol drinking status to examine the genotype-specific cancer risk in each subgroup. Cut-off of age was based on the median age among controls. The significance of the interactions was determined by a likelihood ratio test comparing a full model including the interaction term with the main effect model.

The levels of estradiol in male cases and male controls were compared using multivariable logistic regression model adjusted for age. A square-root transformation was applied to the estradiol concentration. We also assessed the joint association of estradiol level and *ESR2* rs1256049 and rs4986938 genotypes with CRC risk by multivariable logistic regression model. For serum estradiol, the mean estradiol level in all male subjects (including cases and controls) was used as the grouping standard: higher than average concentration was defined as high estradiol level and lower than or equal to average concentration was defined as low estradiol level.

All statistical tests were 2-sided, and a *P* value less than 0.05 was considered to be statistically significant. All analyses were performed using SAS software, version 9.2 (SAS Institute Inc., Cary, NC, USA).

## Results

### Characteristics of participants

Table 1 depicts distributions of selected characteristics of male only patients with CRC and controls. Cases were older, more likely to be never-smokers and never-drinkers than controls in this study. The average age (standard deviation [SD]) at diagnosis of CRC was 58.3 (13.3) years (median, 58 years) in these patients. The average age (SD) at recruitment was 50.0 (9.4) years (median, 48 years) in the healthy controls.

**Table 1:** Selected demographic characteristics of colorectal cancer cases and controls

	**Controls (n = 445)**		**Cases (n = 390)**		
**Characteristics**	**n**	**%**	**n**	**%**	***P*-value**^a^
Age, years					
≤ 48	239	53.7	82	21.0	<0.001
>48	206	46.3	308	79.0	
Smoking status					
Ever	283	63.6	181	46.4	<0.001
Never	162	36.4	209	53.6	
Alcohol drinking Status					
Ever	292	65.6	137	39.0	<0.001
Never	153	34.4	214	61.0	

^a^*P* values were calculated from chi-square test.

### Genotype specific risk association analyses

Two *ESR2* SNPs, rs1256049 and rs4986938, were genotyped. Among controls, genotype distributions were in Hardy-Weinberg equilibrium (*P* = 0.686 for rs1256049 and *P* = 0.669 for rs4986938).

Table 2 shows the genotype specific risks of CRC in the studied population: compared to the homozygous CC genotype, CT/TT genotypes of rs1256049 and rs4986938 were associated with significant reduced and increased risk of CRC, respectively. Similarly, under log-additive model, risk estimates were 0.8 (95% CI, 0.6–1.0) for rs1256049 and 1.5 (95% CI, 1.0–2.0) for rs4986938. No significant association was found for codominant model (data not shown).

**Table 2:** ***ESR2 *genotype frequencies in colorectal cancer cases and controls and odds ratio estimates**

	**Controls**^**a**^**(n = 445)**		**Cases (n = 390)**			
**Genotypes**	**n**	**%**	**n**	**%**	**OR**^**b**^**(95% CI)**	***P*-value**^**b**^
**rs1256049**						
CC (Ref.^c^)	167	37.9	168	43.9	1.0	0.029
CT/TT	274	62.1	215	56.1	0.7 (0.5–1.0)	
**rs4986938**						
CC (Ref.^c^)	349	79.9	277	71.8	1.0	0.036
CT/TT	88	20.1	109	28.2	1.5 (1.0–2.1)	
**rs1256049 + rs4986938**						
0 risk genotype (Ref.^c^)	232	53.2	165	43.4	1.0	0.014
1 risk genotype	156	35.8	154	40.5	1.3 (0.9–1.8)	
2 risk genotypes	48	11.0	61	16.1	2.0 (1.3–3.3)	
					*P*_*trend*_ =	0.003
No risk genotype (Ref.^c^)	232	53.2	165	43.4	1.0	0.016
With risk genotype	204	46.8	215	56.6	1.5 (1.1–2.0)	

^a^ The genotype frequencies in controls were in Hardy-Weinberg equilibrium (*P* = 0.686 for rs1256049 and *P* = 0.669 for rs4986938, Chi-square test).

^b^ORs and *P* values were calculated using unconditional logistic regression model adjusted for age, smoking status and alcohol drinking status.

^c^Ref. = reference group.

In order to assess the joint effect of these polymorphisms on CRC risk in men, we categorized all subjects into 3 groups based on the number of risk genotypes which were determined on the basis of the risk estimates in Table 2. The risk genotypes were rs1256049 CC genotype and rs4986938 CT/TT genotypes. We also dichotomized patients into groups with and without risk genotype. As shown in Table 2, subjects with risk genotype had a significantly increased risk of CRC compared with those carrying no risk genotype (OR, 1.5, 95% CI, 1.1–2.0). Specifically, subjects carrying risk genotype of both polymorphisms had the highest risk of developing CRC (OR, 2.0, 95% CI, 1.3–3.3). We also found the CRC risk in men increased with the number of risk genotype in a dose–response manner (*P*_*trend*_, 0.003). Additionally, we evaluated the combined effect of risk alleles of rs1256049 and rs4986938 and found increased risk for CRC associated with cumulative number of risk alleles (OR, 1.3, 95% CI, 1.1–1.6, *P*, 0.003).

### Stratification analysis

The genotype specific risks of CRC stratified by age, smoking and alcohol drinking status are shown in Table 3. The protective effect of rs1256049 CT/TT genotypes on CRC was likely confined to old subjects, ever-smokers and ever-drinkers: rs1256049 CT/TT genotypes were associated with significantly reduced risk of CRC in >48 years old subjects (OR, 0.7, 95% CI, 0.5–1.0, *P,* 0.047), in ever-smokers (OR, 0.5, 95% CI, 0.4–0.8, *P,* 0.005), and in ever-drinkers (OR, 0.6, 95% CI, 0.4–1.0, *P,* 0.038), after adjustment for potential confounding factors, while no significant interaction was found with age, smoking status and alcohol drinking status. For rs4986938, the ORs for CT/TT genotypes compared to CC genotype were significant for never-smokers only (OR, 2.2, 95% CI, 1.3–4.0, *P,* 0.005).

**Table 3:** **Stratification analysis of colorectal cancer risk associated with *ESR2 *genotypes**

	**rs1256049**				**rs4986938**	
	**CC (Ref.**^**a**^**)**	**CT/TT**		**CC (Ref.**^**a**^**)**	**CT/TT**	
**Subgroups**	**Ca/Co**^**b**^	**Ca/Co**^**b**^	**OR**^**c**^**(95% CI)**	**Ca/Co**^**b**^	**Ca/Co**^**b**^	**OR**^**c**^**(95% CI)**
Age, years						
≤ 48	30/88	50/150	1.0 (0.5–1.8)	55/186	27/49	1.4 (0.7–2.7)
>48	138/79	165/124	0.7 (0.5–1.0)	222/163	82/39	1.5 (0.9–2.3)
*P*_*interaction*_			0.483			0.831
Ever	88/104	87/175	0.5 (0.4–0.8)	139/226	40/53	1.0 (0.6–1.7)
Never	80/63	128/162	1.0 (0.6–1.6)	138/123	69/35	2.2 (1.3–4.0)
*P*_*interaction*_			0.061			0.058
Alcohol drinking status						
Ever	66/112	66/178	0.6 (0.4–1.0)	100/229	34/57	1.6 (0.9–2.7)
Never	89/123	55/96	0.8 (0.5–1.2)	148/120	66/31	1.5 (0.9–2.4)
*P*_*interaction*_			0.473			0.651

^a^Ref. = reference group.

^b^Ca/Co = colorectal cancer cases/controls.

^c^ORs were calculated using unconditional logistic regression model adjusted for age, smoking status and alcohol drinking status.

### Serum estradiol concentrations in male patients with CRC and controls

**Figure 1:**
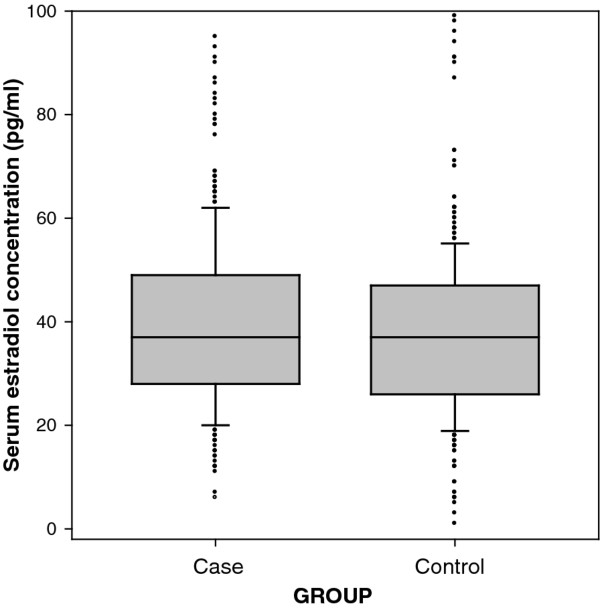
**Serum estradiol concentration in male colorectal cancer cases and controls.** The dots, error bars and upper and lower ends of the box represent outliers, spread, and first and third quartiles, respectively. The line the box represent median.

As illustrated in Figure 1, we observed higher levels of serum estradiol in cases than in controls. Mean (SD) estradiol concentrations were 39.55 (16.25) pg/ml in male patients with CRC and 37.45 (15.86) pg/ml in male controls. Serum estradiol levels were significantly associated with increased risk of CRC in men after adjustment for age; the adjusted OR for serum estradiol levels (continuous) was 1.2 (95% CI, 1.0–1.3,* P *,0.009).

### Joint association of estradiol level and *ESR2* genotypes with the risk of CRC

In Table 4, we combined the estradiol levels and *ESR2* genotypes to estimate their joint effect on risk of male CRC using two models, one adjusted only for age, and another adjusted for potential risk factors of CRC including age, smoking and alcohol drinking status. Using the group of individuals with rs4986938 CC genotype and low level (≤38.47 pg/ml) of estradiol as reference group, the group of individuals with CT/TT genotypes and high level (>38.47 pg/ml) of estradiol had the highest OR of 2.3 (95% CI, 1.4–3.9) after adjusted for age among all subgroups. Such an association was weakened but remained in the same direction after further adjustment with other factors including smoking and alcohol drinking status (OR, 1.6, 95% CI, 0.9–2.9). However, the associations of such combined serum estradiol and *ESR2* genotypes with CRC risk were almost similar among those subgroups for rs1256049.

**Table 4:** **Joint effect of serum estradiol level and *ESR2 *genotypes on colorectal cancer risk in men**

		**Controls (n = 378)**		**Cases (n = 359)**			
**Variables**		**n**	**%**	**n**	**%**	**OR**^**a**^**(95% CI)**	**OR**^**b**^**(95% CI)**
**Estradiol + rs1256049**							
Low	CT/TT	127	33.8	96	27.3	1.0	1.0
	CC	80	21.3	87	24.7	1.6 (1.0–2.4)	1.7 (1.1–2.6)
High	CT/TT	109	29.0	101	28.7	1.5 (1.0–2.3)	1.2 (0.8–1.9)
	CC	60	16.0	68	19.3	1.6 (1.0–2.5)	1.4 (0.8–2.3)
**Estradiol + rs4986938**							
Low	CC	162	43.4	144	40.6	1.0	1.0
	CT/TT	42	11.3	43	12.1	1.1 (0.7–1.9)	1.1 (0.6–1.9)
High	CC	136	36.5	113	31.8	1.0 (0.7–1.4)	0.8 (0.6–1.2)
	CT/TT	33	8.9	55	15.5	2.3 (1.4–3.9)	1.6 (0.9–2.9)

^a^ORs were calculated using unconditional logistic regression model adjusted for age.

^b^ORs were calculated using unconditional logistic regression model adjusted for age, smoking status and alcohol drinking status.

## Discussion

To the best of our knowledge, the present study is the first to explore the potential role of endogenous estrogen on risk of CRC in men. We found serum estradiol level to be positively associated with increased risk of CRC in men. Our study is also the first to evaluate the relationship between *ESR2* rs1256049 and rs4986938 and risk of CRC in Han Chinese and found both SNPs were associated with susceptibility to CRC in men. We observed a significant association between the risk genotypes of these two SNPs in combination and CRC risk, which was statistically significant in a dose–response manner. In addition, men possessing the risk genotypes of rs4986938 and high level of serum estradiol had significant increased risk for developing CRC.

The role of estrogens in CRC risk remains controversial. In the WHI trial, postmenopausal women who randomly assigned to combination of estrogen plus progestin therapy group were at reduced risk for CRC compared to those assigned to placebo group, the reduction (37%) was nominally significant [12]. An early meta-analysis also reported a 20% reduction in risk of colon cancer and 19% reduction in risk of rectal cancer associated with postmenopausal hormone therapy, and particularly current hormone use (34% reduction of CRC risk) [10]. These observations strongly supported an inverse association between exogenous postmenopausal estrogens and CRC risk. However, endogenous estrogens may have an opposite effect on CRC risk. In the nested CRC case–control study from the WHI cohort, circulating estradiol concentration was positively associated with increased risk of CRC [13]. Similarly, the New York University Women’s Health study reported a 1.8-fold increase of CRC risk in the postmenopausal women having the highest quartile of circulating estrone level compared with those having lowest quartile [14]. Although the exact mechanisms are unclear, several observations may help explain the disparate results. First, in the WHI clinical trial, estrogen plus progestin exerted a protective role, whereas oral estrogen alone achieved no such effect [31]. Second, the use of oral estrogens usually couples with increased expression of sex-hormone-binding globulin, which leads to reduced concentrations of bioavailable estrogen [32]. Finally, oral estrogens showed a negative correlation with the synthesis and activity of insulin-like growth factor I axis components, which have a protective role against CRC tumorigenesis [13]. In our study, in agreement with these previous results, we found that high circulating estradiol concentrations were associated with increased risk of CRC in men, supporting the risk effect of endogenous estrogens on CRC development. Further studies to evaluate the sex-difference and dose-difference effect of endogenous estrogens on CRC tumorigensis are warranted.

One of the most intriguing findings in our study was the joint effect of *ESR2* rs4986938 genotype and estradiol levels on CRC risk. Although there was no significant interaction between rs4986938 genotypes and serum estradiol levels for CRC risk in our study (data not shown), the joint effect was likely close to the expected additive scale, that is, individuals with both high estradiol level and rs4986938 risk genotypes had a significant OR of 2.3, compared with individuals with low estradiol level and wild-type genotype, which was higher than the sum of ORs in those with high estradiol level only (OR, 1.1) and in those with wild-type genotype alone (OR, 1.0). This result supports the possible influence of interaction between *ESR2* gene and endogenous estrogens on the risk of CRC. It is noteworthy that the biological action of estrogens has to be through its receptors, and colon tissue is characterized by a predominance of ERβ, the protein encoded by *ESR2*[16]. Although the mechanisms remain largely unknown, frequent loss of *ESR2* expression in CRC tissue has been observed, suggesting involvement of ERβ in development and progression of CRC [17,18,33]. Moreover, some *ESR2* variants have been shown to alter the function of the receptor, affecting the tissue’s response to estrogens [34]. There is also evidence that rs4986938 could affect RNA stability of the *ESR2* gene [35,36]. Our findings of the joint effect of rs4986938 and estradiol levels on CRC risk and the significant associations between *ESR2* SNPs and CRC risk provides supporting evidence of functional potential of *ESR2* gene in estrogen-related CRC tumorigenesis, while *in vivo* and *in vitro* research as well as evaluation in larger population are required for further elucidation.

*ESR2* rs1256049 CT/TT genotypes conferred a reduced risk of CRC in this Han Chinese case–control population. This is in agreement with the study of Slattery et al. which evaluated *ESR2* rs1256049 in a case–control population of vast majority of Caucasian and found that the C allele of rs1256049 conferred an increased risk of rectal cancer among the total population if diagnosed before 60 years of age, and an increased risk in colon cancer if estrogen positive [25]. A study in Japanese population, however, found an increased risk of CRC linked with rs1256049 TT genotype [29]. The ethnic difference may lead to the discrepancy among these studies. More large-scale studies in different ethnic background population are warrant to validate the potential role of *ESR2* rs1256049 in susceptibility to CRC.

There are certain limitations to our findings. First, because all patients enrolled in this study were ethnic Han Chinese, our ability to adequately assess risk in other ethnic populations is limited. Further studies using sufficient numbers of subjects from other races and ethnic groups are needed to extend and confirm our findings. Second, only a limited number of candidate SNPs were selected in this study, which is not fully representative of the complexity of genes in the estrogen receptor pathway. Finally, due to the nature of hospital-based case–control study design, a potential selection bias should be taken into consideration when interpreting the results. One of the reasons for the difference between cases and controls in smoking status could be the selection bias. Additionally, using hospital-based controls could generate Berkson bias which might influence the frequencies of *ESR2* genotypes and the susceptibility to CRC risk. However, the genotype frequencies in the controls did not deviate significantly from the Hardy-Weinberg equilibrium, thus, the selection bias may have been minimized in this study.

## Conclusions

Our findings may support a role for estrogens in colorectal tumorigenesis. We found that both serum estradiol and *ESR2* genetic variants individually were associated with risk of CRC. In addition, individuals with both rs4986938 risk genotypes and high estradiol level had a higher risk for developing CRC in men. Therefore, the functional SNPs, hormone environmental factors, and their interactions, may provide a new clue for individualized evaluation CRC and offer effective prevention strategies in the future. However, our findings need to be validated by larger studies, and additional functional investigations of mechanisms are warranted.

## Abbreviations

CI: Confidence interval; CRC: Colorectal cancer; ER: Estrogen receptor; ESR2: Estrogen receptor β gene; OR: Odds ratio; SD: Standard deviation; SNP: Single nucleotide polymorphism; WHI: Women’s Health Initiative.

## Competing interests

The authors declare that they have no competing interests.

## Authors’ contributions

HW collected the data, performed the experiment and drafted the manuscript. LX performed data analysis and drifted the manuscript. JC, JB, SY, GH, and LH participated in patients’ recruitment, study materials collection and experiment design. XC, XY and GL participated in the design of the study, interpretation of data and revision of the manuscript. All authors read and approved the final manuscript.

## Pre-publication history

The pre-publication history for this paper can be accessed here:

http://www.biomedcentral.com/1471-2407/12/276/prepub
